# Optimized split nitrogen fertilizer increase photosynthesis, grain yield, nitrogen use efficiency and water use efficiency under water-saving irrigation

**DOI:** 10.1038/s41598-020-75388-9

**Published:** 2020-11-20

**Authors:** Zhen Zhang, Yongli Zhang, Yu Shi, Zhenwen Yu

**Affiliations:** Key Laboratory of Crop Ecophysiology and Farming System, Ministry of Agriculture, Shandong Agricultural University, Taian, 271017 Shandong China

**Keywords:** Photosynthesis, Field trials

## Abstract

This study aims to investigate optimization of the basal-top-dressing nitrogen ratio for improving winter wheat grain yield, nitrogen use efficiency, water use efficiency and physiological parameters under supplemental irrigation. A water-saving irrigation (SI) regime was established and sufficient irrigation (UI) was used as a control condition. The split-nitrogen regimes used were based on a identical total nitrogen application rate of 240 kg ha^−1^ but were split in four different proportions between sowing and the jointing stage; i.e. 10:0 (N1), 7:3 (N2), 5:5 (N3) and 3:7 (N4). Compared with the N1, N2 and N4 treatments, N3 treatment increased grain yield, nitrogen and water use efficiencies by 5.27–17.75%, 5.68–18.78% and 5.65–31.02%, respectively, in both years. The yield advantage obtained with the optimized split-nitrogen fertilizer application may be attributable to greater flag leaf photosynthetic capacity and grain-filling capacity. Furthermore, the N3 treatment maintained the highest nitrogen and water use efficiencies. Moreover, we observed that water use efficiency of SI compared with UI increased by 9.75% in 2016 and 10.79% in 2017, respectively. It can be concluded that SI along with a 5:5 basal-top-dressing nitrogen ratio should be considered as an optimal fertigation strategy for both high grain yield and efficiency in winter wheat.

## Introduction

The North China Plain (NCP) is a major wheat producing area in China which accounts for 25% of the country’s total farm land and contributes 71% of the total wheat production^1,2^. However, the rainfall in this region is insufficient to meet winter wheat requirements, as only 100–180 mm of precipitation falls during the winter wheat growing season, which is approximately 30% of the annual precipitation^3,4^. Wheat is accordingly irrigated using water extracted from groundwater sources, which has led to a detrimental lowering of the groundwater table by approximately 1 m year^−1^ over the last 20 years^5^. In this background, and after years of experiments, we established a wheat water-saving cultivation technique based on supplementary irrigation determined by measuring soil moisture, which reduces irrigation input and ensures high yields and water use efficiency^6,7^.

With regards to wheat cultivation, nitrogen, as an essential macronutrient, is required more consistently and in larger amounts than any other nutrient^8^. Under application of nitrogen fertilizer in wheat production can prolong the grain-filling stage and improve photosynthesis capacity, thereby enhancing grain yield^9^. However, over nitrogen application has been found to result in an increase in nitrogen loss and decreases in nitrogen use efficiency and grain yield^10,11^. Previous studies have shown that total application of nitrogen as a basal fertilizer results not only in high nitrogen loss via volatilization but also low nitrogen utilization efficiency, and that top-dressing applications can enhance grain yield and nitrogen use efficiency compared with exclusive basal applications^12^. Furthermore, some studies have shown that under conditions in which the total amount of nitrogen applied is 202.5 kg ha^−1^, the highest grain yields obtained using a treatment with a 4:4:2 ratio applied at the sowing, jointing, anthesis stages were 11.01% and 9.60% higher than those of treatments with a ratio of 6:4 and 4:6 applied at sowing and the joining stage^13^.

**Figure 1:**
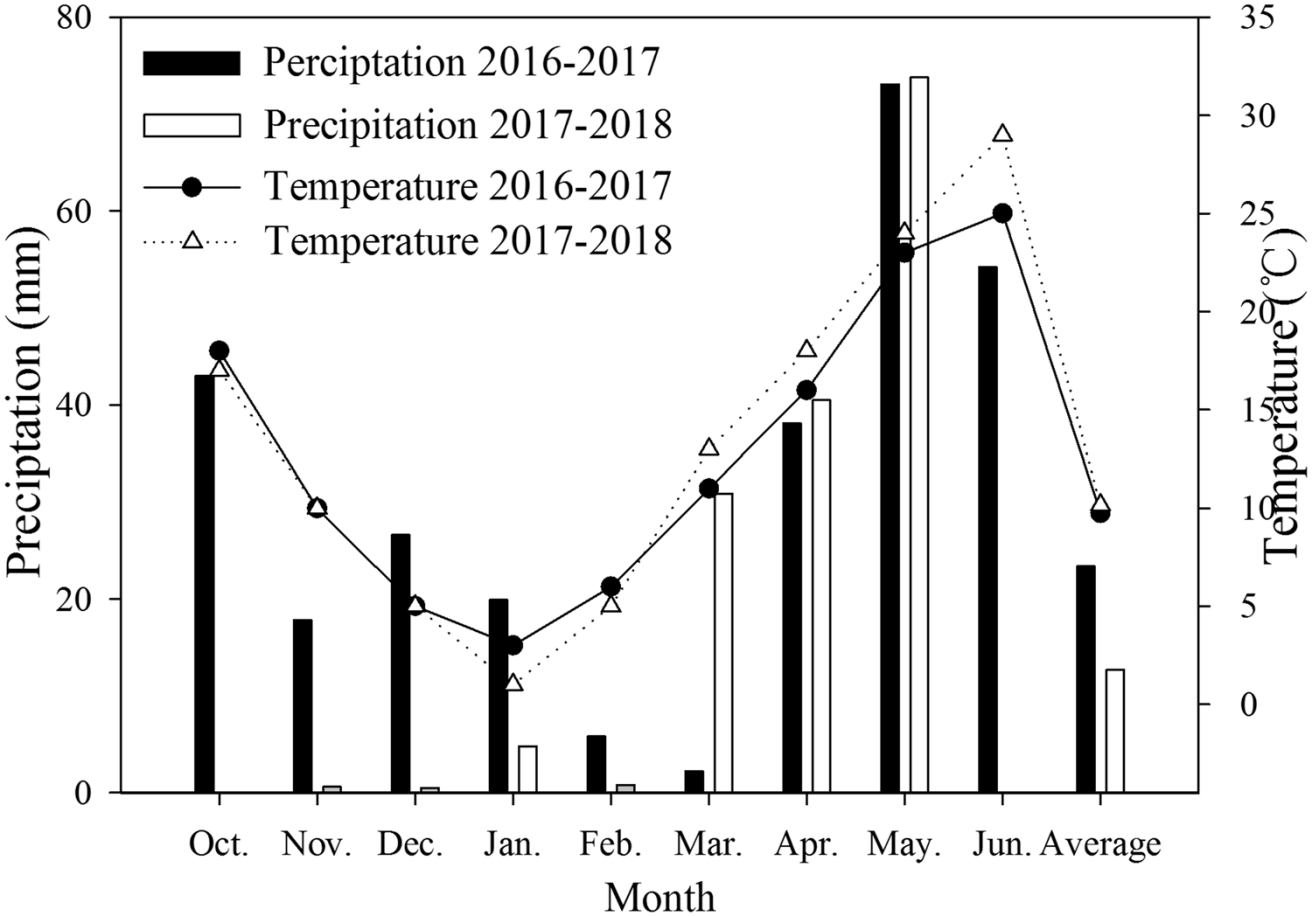
Effective precipitation and temperature during wheat growth period.

However, nitrogen fertilizer management measures are inevitably affected by irrigation^14^. Soil moisture can facilitate the diffusion of soil dissolved inorganic nitrogen and promote a better contact of soil water with the wheat root system to enhance water and nitrogen use efficiencies^15,16^. And in turn, nitrogen fertilizer can contribute to increasing wheat water productivity^17^. It has been reported that, interaction of water and nitrogen are the main factors that affect yield and resource use efficiency of wheat production^18^. Various irrigation and nitrogen fertilizer management practices have been suggested for wheat production including water-saving irrigation, reduced nitrogen fertilizer level and a combination of chemical and organic fertilizers^19^. However, although previous studies have investigated the effects of nitrogen management under quantitative irrigation on photosynthetic capacity and grain yield, there is limited information regarding the optimization of split-nitrogen management under the water-saving technique of supplemental irrigation based on measuring soil moisture, and thus further investigations are needed^20,21^. In this context, we used a identical total nitrogen rate under supplemental irrigation based on measuring soil moisture, although with the applied nitrogen split into different proportions between basal and top-dressings at the jointing stage, to assess which proportion was optimal under two supplemental irrigation conditions. We conducted a 2-year field experiment to assess whether the photosynthetic performance of flag leaves and grain-filling capacity of wheat after anthesis under water-saving irrigation conditions were improved in response to supplemental irrigation and split nitrogen application. Accordingly, the objectives of this study were as follows: to evaluate the effect of split-nitrogen management on the photosynthetic performance of flag leaves and the grain-filling capacity under water-saving irrigation conditions in the NCP (Fig. 1).

## Results

### Photosynthetic parameters of flag leaves after anthesis

**Figure 2:**
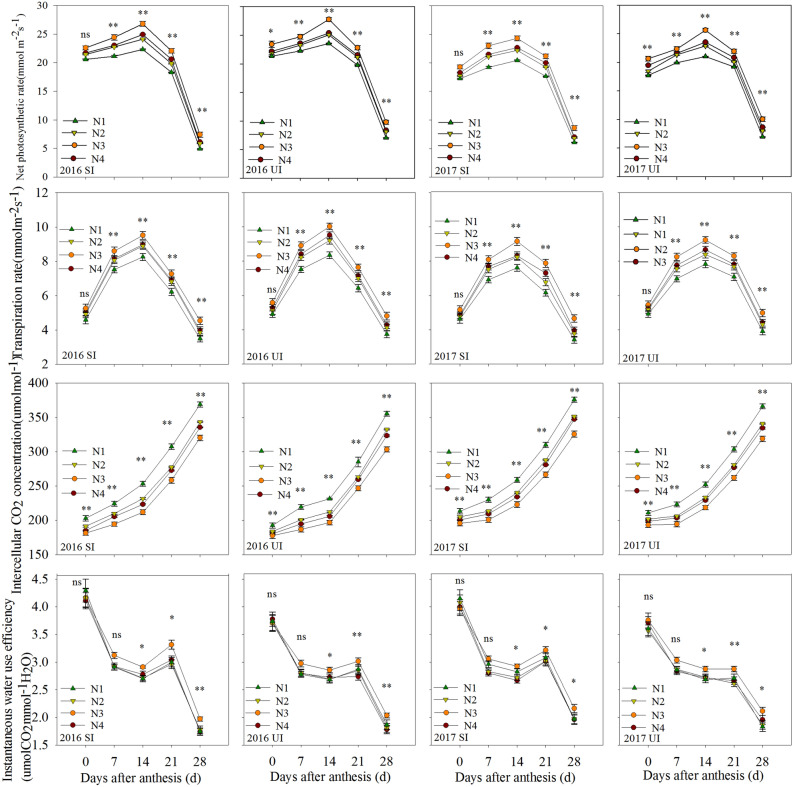
Effects of different treatments on photosynthetic capacity of flag leaves after anthesis. *^,^**Significant at the 0.05 and 0.01 probability levels, respectively, *ns* no significant.

We found that the Pn, Tr, Ci and IWUE of the flag leaves from wheat after anthesis were significantly different among split nitrogen treatments (Table 1). Compared with N1, N2 and N4 treatments, there were significant increases in Pn, Tr and IWUE, but a decreased in Ci in response to N3 treatment at 7, 14, 21 and 28 days after anthesis under water-saving treatment (Fig. 2). A similar trend was observed under the sufficient irrigation treatment. A comparison of Pn, Tr and Ci showed no significantly differences among water-saving and sufficient irrigations. However, the water-saving irrigation treatment increased IWUE by 5.34% in 2016 and 6.45% in 2017, compared with the sufficient irrigation treatment (Fig. 2). The significant interaction effect between nitrogen fertilizer management and irrigation levels showed that their interaction had no significant effects on photosynthetic parameters of flag leaves (Table 1). Moreover, the growth year had significant effects on the Pn, Tr, Ci and IWUE of flag leaves after anthesis (Table 1).

**Table 1:** F-test of nitrogen treatment, irrigation treatment, year, nitrogen × irrigation, nitrogen × year, irrigation × year, and nitrogen × year × irrigation interactions on photosynthetic capacity of flag leaves in winter wheat under different nitrogen and irrigation treatments.

Source	Net photosynthetic	Transpiration rate	Intercellular CO_2_ concentration	Instantaneous water use efficiency
Nitrogen (N)	**	**	**	**
Irrigation (I)	ns	ns	ns	*
Year (Y)	**	**	*	*
N × I	ns	ns	ns	ns
N × Y	ns	ns	ns	ns
I × Y	*	*	**	**
N × I × Y	ns	*	ns	**

*^,^**Significant at the 0.05 and 0.01 probability levels, respectively, ns, no significant.

### Chlorophyll fluorescence parameters of flag leaves

We also found that the chlorophyll fluorescence parameters Fv/Fm, ΦPSǁ, Qp and ETR of flag leaves were significantly affected by the split-nitrogen treatments (Table 2). Under water-saving and sufficient irrigation treatments, there were significant increases in Fv/Fm, ΦPSǁ, Qp and ETR in response to the N3 treatment compared with the N1, N2 and N4 treatments (Table 2). However, we detected no significant difference between the water-saving and sufficient irrigation treatments with respect to Fv/Fm, ΦPSǁ, Qp and ETR. But their interaction (N × I) had no significant effects on Chlorophyll fluorescence parameters of flag leaves (Table 2). The growth year also had significant effects on Fv/Fm, ΦPSǁ, Qp and ETR , which were all higher in 2016 than in 2017 (Table 2).

**Table 2:** Effects of different treatments on fluorescence parameters after anthesis.

Treatments	Fv/Fm		ΦPSǁ		Qp		ETR	
	2016	2017	2016	2017	2016	2017	2016	2017
**SI**								
N1	0.712c	0.673c	0.586c	0.541c	0.644c	0.541c	0.246c	0.227c
N2	0.768b	0.715b	0.624b	0.579b	0.692b	0.579b	0.261b	0.243b
N3	0.814a	0.757a	0.704a	0.621a	0.744a	0.626a	0.281a	0.261a
N4	0.772b	0.718b	0.635b	0.588b	0.705b	0.588b	0.267b	0.247b
**UI**								
N1	0.731c	0.682c	0.594c	0.554c	0.654c	0.554c	0.250c	0.233c
N2	0.784b	0.726b	0.633b	0.597b	0.704b	0.597b	0.266b	0.251b
N3	0.838a	0.773a	0.677a	0.634a	0.752a	0.638a	0.285a	0.266a
N4	0.792b	0.734b	0.643b	0.603b	0.714b	0.603b	0.270b	0.253b
**ANOVA**								
Nitrogen (N)	**		**		**		**	
Irrigation (I)	ns		ns		ns		ns	
Year (Y)	**		*		**		*	
N × I	ns		ns		ns		ns	
N × Y	ns		ns		ns		ns	
I × Y	ns		ns		ns		ns	
N × I × Y	ns		ns		ns		ns	

Different small letters in the same column indicate significant differences at the 5% level.

*^,^**Significant at the 0.05 and 0.01 probability levels, respectively, ns, no significant.

Taken together, the aforementioned comparisons indicated that the 5:5 basal-top-dressing nitrogen ratio increased the Pn, Tr, IWUE, Fv/Fm, ΦPSǁ, Qp and ETR of wheat flag leaves after anthesis under both the water-saving and sufficient irrigation treatments. Moreover, a comparison of the photosynthetic parameters of flag leaves showed no significantly differences among water-saving and sufficient irrigations for most measurements. An exception, however, was the IWUE in both years, with the increase in IWUE under the water-saving irrigation treatment being significantly higher than that under sufficient irrigation.

### 1000-grain weight

**Figure 3:**
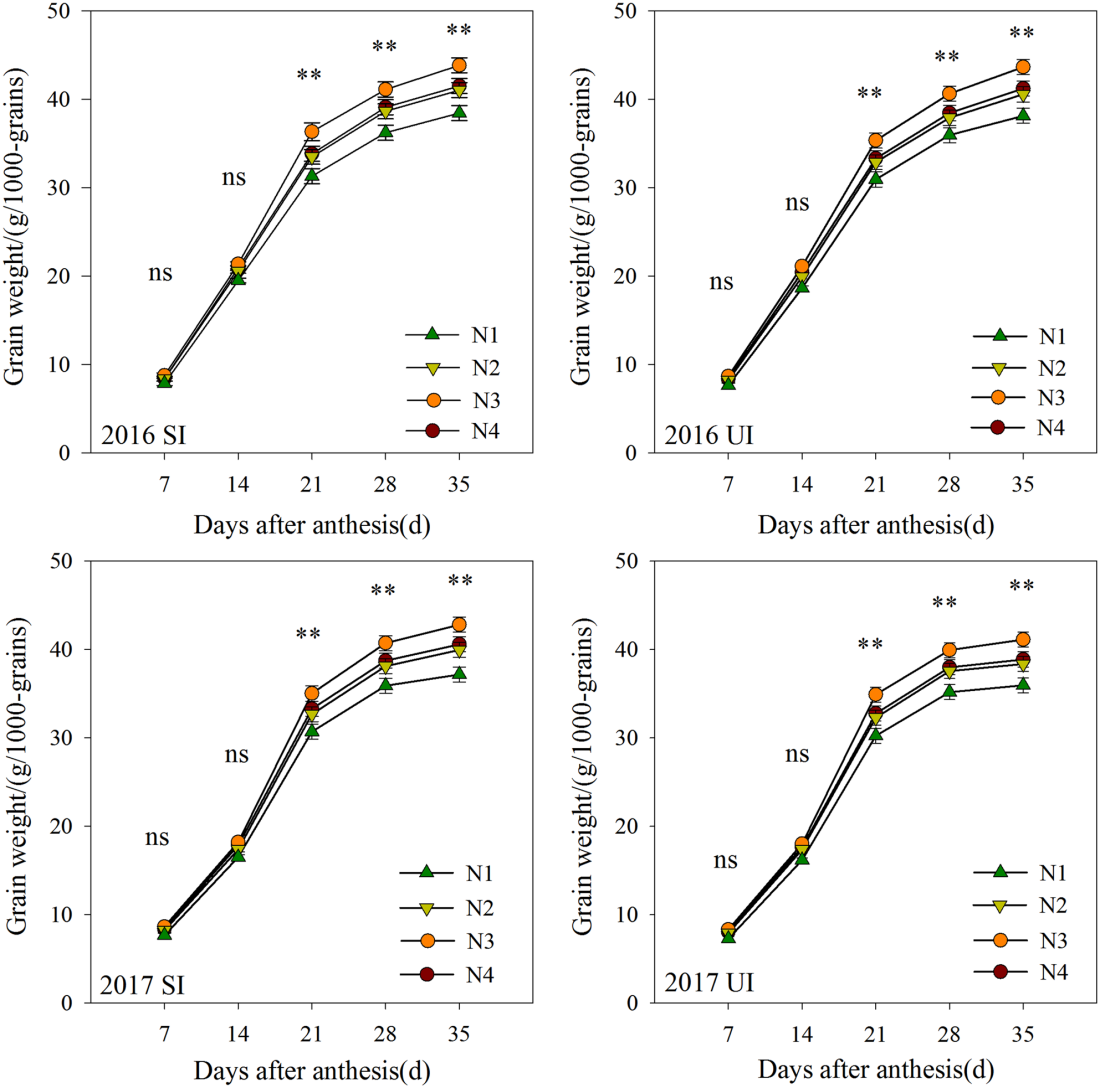
Effects of different treatments on grain weight after anthesis. Different small letters in the same column indicate significant differences at the 5% level. *^,^**Significant at the 0.05 and 0.01 probability levels, respectively, *ns* no significant.

As shown in Fig. 3, the 1000-grain weight of wheat increased gradually with an increase in the number of days after anthesis, and reached a peak value at 35 days. Under both water-saving and sufficient irrigation treatments, significant differences in the 1000-grain weight were observed between the different split-nitrogen treatments at 21, 28 and 35 days after anthesis. The 1000-grain weight under the N3 treatment reached 36.94, 41.13, 43.81 and 35.37, 40.66, 43.68 g at 21, 28 and 35 days after anthesis in the 2016 and 2017 growth seasons, respectively, which were higher than those obtained under the N1, N2 and N4 treatments. In contrast, we observed no significant differences among split-nitrogen treatments with respect to the 1000-grain weight values measured at 7 and 14 days after anthesis. Furthermore, there were no significant differences between the water-saving and sufficient irrigation treatments in this regard.

### Grain filling

Table 3 shows the grain-filling parameters. We found that the split-nitrogen treatments and supplemental irrigation had significant effects on Vmax and Vmean values, although did not significantly affect Tmax or D. And their interaction (N × I) had no significant effects on the grain-filling parameters. Under both water-saving and sufficient irrigation treatments, the Vmax and Vmean in response to the N3 treatment were significantly higher than those of the N1 treatment, whereas we detected no significant differences between the N3 and N2 treatments or the N3 and N4 treatments. Moreover, we found that the increases in Vmax and Vmean values recorded under the water-saving irrigation treatment were significantly higher than those obtained under the sufficient irrigation treatment. The growth year also had significant effects on Vmax, although not Vmean, Tmax or D.

**Table 3:** Grain filling equation and grouting parameters for different treatments.

Year	Treatments	Growth curve equation	Tmax (d)	Vmax (mg grain^−1^ d^−1^)	Vmean (mg grain^−1^ d^−1^)	D (d)
2016	**SI**					
	N1	y = 38.8454/(1 + 15.7128e−0.1977x)	13.93a	1.92b	1.03b	30.35a
	N2	y = 41.4779/(1 + 16.1018e−0.1986x)	13.99a	2.06a	1.11a	30.21a
	N3	y = 44.3454/(1 + 15.8015e−0.1927x)	14.32a	2.14a	1.15a	31.13a
	N4	y = 41.9348/(1 + 16.0675e−0.1987x)	13.97a	2.08a	1.12a	30.19a
	**UI**					
	N1	y = 38.6580/(1 + 16.5898e−0.1975x)	14.22a	1.91b	1.02b	30.38a
	N2	y = 41.0012/(1 + 16.0411e−0.1960x)	14.16a	2.01a	1.08a	30.61a
	N3	y = 44.1048/(1 + 14.9808e−0.1872x)	14.46a	2.06a	1.12a	32.04a
	N4	y = 41.6409/(1 + 15.5419e−0.1944x)	14.11a	2.02a	1.09a	30.86a
2017	**SI**					
	N1	y = 38.1800/(1 + 19.7060e−0.2022x)	14.74a	1.93b	1.01b	29.68a
	N2	y = 40.9641/(1 + 19.3350e−0.1989x)	14.89a	2.04a	1.07a	30.16a
	N3	y = 44.1439/(1 + 18.4661e−0.1909x)	15.28a	2.11a	1.11a	31.43a
	N4	y = 41.6026/(1 + 18.9175e−0.1985x)	14.81a	2.06a	1.09a	30.22a
	**UI**					
	N1	y = 36.9862/(1 + 21.6040e−0.2000x)	15.36a	1.85b	0.96b	30.00a
	N2	y = 39.5136/(1 + 20.8146e−0.1982x)	15.32a	1.96a	1.02a	30.27a
	N3	y = 42.3890/(1 + 20.4515e−0.1928x)	15.65a	2.04a	1.06a	31.12a
	N4	y = 39.9975/(1 + 20.6329e−0.1982x)	15.27a	1.98a	1.03a	30.27a
**ANOVA**						
Nitrogen (N)			ns	**	**	ns
Irrigation (I)			ns	**	**	ns
Year (Y)			ns	**	ns	ns
N × I			ns	ns	ns	ns
N × Y			ns	ns	ns	ns
I × Y			ns	*	**	*
N × I × Y			ns	ns	ns	ns

Tmax, the time to reach the maximum filling rate; Vmax, maximum filling rate; Vmean, mean filling rate; D, grain filling duration.

*^,^**Significant at the 0.05 and 0.01 probability levels, respectively, ns: no significant.

Correlation analysis revealed that the 1000-grain weight was positively correlated with the duration of maximum grain-filling rate, maximum grain-filling rate and average grain-filling rate, but was negatively correlated with the duration of grain filling (Table 4).

**Table 4:** Correlation analysis between 1000-grain weight and grain filling parameters.

	Tmax	Vmax	Vmean	D
1000-grain weight	0.948**	0.871**	0.978**	− 0.668

Tmax, the time to reach the maximum filling rate; Vmax, maximum filling rate; Vmean, mean filling rate; D, grain filling duration.

**Significant at the 0.01 probability levels.

### Grain potential storage capacity (GPSC) and storage capacity index (SCI)

As shown in Table 5, the split-nitrogen treatments, supplemental irrigation treatments and their interaction (N × I) had significant effects on GPSC and SCI. Under both water-saving and sufficient irrigation treatments, the N3 treatment significantly increased GPSC and SCI, compared with N1, N2 and N4 treatments. Furthermore, in response to the water-saving irrigation treatment, GPSC and SCI were increased by 5.80%, 6.90% in 2016 and 5.85%, 7.33% in 2017, respectively, compared with the sufficient irrigation treatment. Although the growth year had no significant effects on GPSC, significant effects were observed with respect to SCI.

**Table 5:** The effects of different treatments on GPSC, SCI were also studied.

Treatments	GPSC (L)		SCI (%)	
	2016	2017	2016	2017
**SI**				
N1	85.69c	84.83c	88.15c	82.82c
N2	90.19b	89.75b	93.16b	89.59b
N3	93.92a	94.62a	98.46a	94.73a
N4	89.77b	90.02b	94.96b	89.25b
**UI**				
N1	80.86c	79.47c	84.12c	77.43c
N2	85.09b	85.31b	87.80b	82.50b
N3	88.21a	88.07a	90.63a	87.54a
N4	85.66b	86.55b	87.98b	84.58b
**ANOVA**				
Nitrogen (N)	**		**	
Irrigation (I)	**		**	
Year (Y)	ns		**	
N × I	*		**	
N × Y	ns		ns	
I × Y	ns		*	
N × I × Y	ns		*	

GPSC, grain potential storage capacity; SCI, storage capacity index; Different small letters in the same column indicate significant differences at the 5% level.

*^,^**significant at the 0.05 and 0.01 probability levels, respectively. ns, no significant.

### Plant nitrogen concentration at different growth stages

As shown in Table 6, under both water-saving and sufficient irrigation treatments, the split-nitrogen treatments had no significant effect on plant nitrogen concentration at before-winter stage. But the N3 treatment significantly increased plant nitrogen concentration at jointing, anthesis and maturity stages. And we also detected no significant differences were observed between the two irrigation treatments. Furthermore, the growth year had no significant effects on plant nitrogen concentration.

**Table 6:** The effects of different treatments on plant nitrogen concentration at different growth stages.

Treatments	Plant nitrogen concentration (%)							
	Before-winter		Jointing		Anthesis		Maturity	
	2016	2017	2016	2017	2016	2017	2016	2017
**SI**								
N1	3.62a	3.56a	2.62a	2.45a	1.50c	1.46c	1.37c	1.31c
N2	3.59a	3.58a	2.58a	2.33a	1.67b	1.59b	1.50b	1.40b
N3	3.66a	3.51a	2.59a	2.38a	1.81a	1.72a	1.59a	1.53a
N4	3.68a	3.52a	2.44b	2.29b	1.70b	1.62b	1.49b	1.42b
**UI**								
N1	3.64a	3.58a	2.63a	2.45a	1.49c	1.45c	1.36c	1.30c
N2	3.63a	3.62a	2.61a	2.44a	1.69b	1.61b	1.52b	1.41b
N3	3.76a	3.60a	2.66a	2.45a	1.87a	1.78a	1.65a	1.60a
N4	3.72a	3.57a	2.47b	2.31b	1.73b	1.65b	1.51b	1.44b
**ANOVA**								
Nitrogen (N)	ns	ns	*	*	**	**	**	**
Irrigation (I)	ns	ns	ns	ns	ns	ns	ns	ns
Year (Y)	ns	ns	ns	ns	ns	ns	ns	ns
N × I	ns	*	ns	ns	ns	*	ns	ns
N × Y	*	ns	ns	ns	ns	ns	ns	ns
I × Y	ns	ns	ns	ns	ns	ns	ns	ns
N × I × Y	ns	ns	ns	ns	ns	ns	ns	ns

*^,^** significant at the 0.05 and 0.01 probability levels, respectively, ns, no significant.

### Grain yield (GY), nitrogen use efficiency (NUE), and water use efficiency (WUE)

The results of ANOVA indicated significant differences among the grain yields obtained under the four different split-nitrogen treatments, whereas there were no significant differences with regards to irrigation treatment (Table 7). And their interaction (N × I) had no significant effects on grain yield. Under the water-saving irrigation treatment, N3 significantly increased grain yield by 16.27%, 7.09%, 5.70% in 2016 and by 16.72%, 8.88%, 5.89% in 2017, compared with N1, N2 and N4 treatments, respectively. Similarly, in response to sufficient irrigation, N3 significantly increased grain yield by 17.75%, 10.68%, 5.27% in 2016 and by 15.22%, 8.01%, 6.87% in 2017, compared with the N1, N2 and N4 treatments, respectively. There was also a significant effect of growth on grain yield, which was higher in 2016 than in 2017.

**Table 7:** The effects of different treatments on GY, NUE and WUE were also studied.

Treatments	GY (kg ha^−1^)		NUE (kg kg^−1^)		WUE (kg mm^−1^ ha^−1^)	
	2016	2017	2016	2017	2016	2017
**SI**						
N1	7293.17c	6906.80c	30.39c	28.78c	14.41d	16.19d
N2	7917.93b	7403.94b	32.99b	30.85b	16.84c	18.02c
N3	8479.68a	8061.52a	35.33a	33.59a	18.88a	20.20a
N4	8022.63b	7612.93b	33.43b	31.72b	17.87b	19.12b
**UI**						
N1	7450.25d	7163.88c	30.77c	29.85c	13.69d	14.83d
N2	7926.01c	7642.02b	33.03b	31.84b	14.96c	16.35b
N3	8772.33a	8254.17a	36.55a	34.39a	17.19a	18.16a
N4	8333.01b	7723.31b	34.12b	32.18b	16.12b	17.01b
**ANOVA**						
Nitrogen (N)	**		**		**	
Irrigation (I)	ns		ns		**	
Year (Y)	**		**		**	
N × I	ns		ns		**	
N × Y	ns		ns		**	
I × Y	ns		ns		*	
N × I × Y	**		**		*	

GY, grain yield; NUE, nitrogen use efficiency; WUE, water use efficiency. Different small letters in the same column indicate significant differences at the 5% level.

*^‚^**Significant at the 0.05 and 0.01 probability levels, respectively. ns, no significant.

We also detected significant differences in the NUE obtained under the four split-nitrogen treatments, whereas no significant differences were observed between the two irrigation treatments. And their interaction (N × I) had no significant effects on NUE. Under both irrigation treatments, N3 treatment significantly increased NUE compared with the N1, N2 and N4 treatments (Table 7). The growth year also had significant effects on NUE, which was higher in 2016 than in 2017.

We found that WUE was also significantly differently affected by the different split-nitrogen, supplemental irrigation treatments and their interaction (Table 7). Under both irrigation treatments, wheat treated with the N3 region showed a significantly enhanced WUE compare with the N1, N2 and N4 treatments (Table 7). Furthermore, compared with the sufficient irrigation treatment, the water-saving irrigation treatment increased WUE by 9.75% in 2016 and 10.79% in 2017. The growth year also had significant effects on WUE, which was higher in 2016 than in 2017. Taken together, the grain yield, NUE and WUE of wheat subjected to the water-saving irrigation treatment were all highest in plants treated with a 5:5 ratio of basal-top-dressing nitrogen.

### Correlation analysis of GY, NUE, WUE, photosynthetic capacity and grain-filling parameters

The results of correlation analysis, based on correlation coefficients calculated by compiling the data obtained under different irrigation and nitrogen fertilizer treatments in the growing seasons from 2016 to 2018, are presented Table 8. This analysis revealed that grain yields were significantly negatively correlated with Ci, but significantly positively correlated with other measured parameters. The relationships between NUE and other indices were found to be similar to those observed for grain yield. In contrast, WUE was significantly positively correlated with fluorescence parameters, Vmax and Vmean, whereas no significant correlations were observed for other indices. Collectively, these correlation results indicated that improving photosynthetic performance and promoting grain filling after anthesis are effective approaches for obtaining higher grain yields, nitrogen use efficiency and water use efficiency.

**Table 8:**
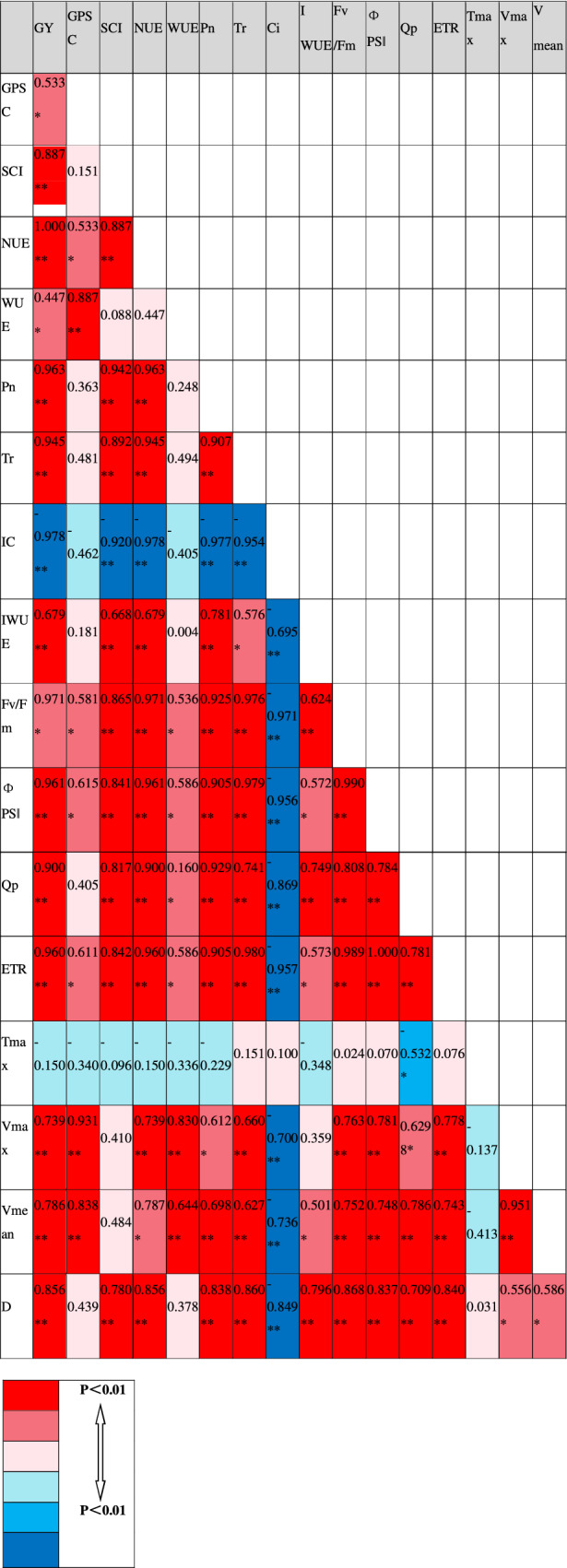
Coefficients of correlation between different indexes at different treatments.

GY, grain yield; GPSC, grain potential storage capacity; SCI, storage capacity index; NUE, nitrogen use efficiency; WUE, water use efficiency; Pn, net photosynthetic rate; Tr, transpiration rate; Ci, intercellular CO_2_ concentration; IWUE, instantaneous wate use efficiency; Fv/Fm, maximum photochemical efficiency; ΦPSǁ, actual photochemical efficiency; Qp, photochemical quenching index; ETR, electron transport rate; Tmax, the time to reach the maximum filling rate; Vmax, maximum filling rate; Vmean, mean filling rate; D, grain filling duration.

*^,^**Significant at the 0.05 and 0.01 probability levels, respectively.

## Discussion

The findings of a number of previous studies have indicated that photosynthetic capacity is the primary factor determining the grain yield of wheat after anthesis^22^. And this capacity is closely related to nitrogen fertilizer application and irrigation^23^. Consistently, in the present study, we demonstrated that under a supplemental water-waving irrigation based on the moisture of soil under wheat cultivation, a 5:5 ratio of basal-top-dressing nitrogen could significantly improve the photosynthetic capacity of flag leaves after anthesis, which proved beneficial in terms of increasing grain filling. This effect could be explained by the fact that a rational basal-top-dressing nitrogen ratio can improve the accumulation of nitrogen in leaves, and that this in turn is significantly positively correlated with chlorophyll activity and enhanced photosynthetic capacity^24^. The increase in Fv/Fm, ΦPSǁ, Qp and ETR of flag leaves after anthesis provides further evidence in support of this assumption. Although previous studies have indicated that adequate irrigation is an important basis for efficient photosynthetic capacity^25^, the findings of the present study indicate that the water-saving and sufficient irrigations had no significant effects on the photosynthetic capacity and chlorophyll fluorescence parameters of flag leaves after anthesis. However, treatment with the optimal combination 5:5 basal-top-dressing nitrogen ratio and water-saving irrigation was associated with the highest photosynthetic capacity and chlorophyll fluorescence parameters. These results reveal that 5:5 basal-top-dressing nitrogen ratio and water-saving irrigation enhances the efficiency of PSII and ETR that could enhance the photosynthesis capacity by improving the energy transport from PSII to PSI. Meanwhile, we assume that a rational basal-top-dressing nitrogen ratio and supplemental irrigation can significantly improve the activities of enzymes in various light energy conversion processes and maintain a high level of activity after anthesis, which might be one of the other primary reasons why the photosynthetic capacity observed under this combined treatment was significantly higher than that obtained with other treatments^26^.

The grain-filling period is a key growth period with regards to increasing the grain weight and yield formation of wheat. Accordingly, enhancing the grain-filling rate represents an effective means of increasing the grain yield of wheat. In this regard, some previous studies have shown that high levels of nitrogen fertilizer have no significant effect on grain weight, or can even reduce grain weight under water deficit conditions^27^. These findings accordingly indicate that water and nitrogen fertilizer have complex effects on the grain weight of wheat^28^. In the present study, we demonstrated that in both growth seasons, a 5:5 basal-top-dressing nitrogen ratio under two levels of supplemental irrigation produced the highest 1000-grains weight. The grain weight advantage obtained by optimizing split-nitrogen fertilizer application may be attributable to a greater grain-filling capacity with higher maximum and mean filling rates. As shown in previous studies, suitable nitrogen fertilizer application has significant effects on the grain-filling capacity of wheat. Moreover, our results revealed that there were no significant differences between the two supplemental irrigation treatments with regards to the parameters of grain-filling and 1000-grains weight. We suspect that inconsistencies with respect to the findings of the present and past studies may be due to the fact that an increase in irrigation can significantly delay the senescence of wheat leaves, and under these conditions, the assimilates stored in vegetative organs are not fully converted into grain yield at grain harvest^29^. These results thus indicate that treatment using a 5:5 basal-top-dressing nitrogen ratio may ensure that a greater amount of assimilates is translocation to the grains.

The response of winter wheat to different combinations of nitrogen application and supplemental irrigation has been the subject of several studies that have detected a positive interaction between suitable nitrogen and irrigation treatments^30^. However, these studies did not examine the effects of split-nitrogen fertilizer application. In the present study, we found that the 5:5 basal-top-dressing nitrogen ratio under two supplemental irrigation treatments produced the highest grain yield in both the growth seasons we assessed. We suspect that this response could be attributed to the fact that splitting the applied nitrogen can significantly increase grain yield via an increase in the photosynthetic capacity of flag leaves and improved grain filling, including increased Pn at the grain-filling stage and leaf fluorescence performance^31^. It is noteworthy that excessive basal and topdressing ratios resulted in lower grain yield. The decrease in yield has two main explanation. The first explanation is that leaf photosynthetic capacity decreased due to insufficient nitrogen supply in late growth season under excessive basal nitrogen fertilizer. The second explanation is that excessive topdressing nitrogen fertilizer disrupts normal plant senescence, leading to postponed grain-filling and maturity.

Appropriate irrigation and split-nitrogen management are helpful to improve the nitrogen use efficiency of wheat. In our study, our results suggested that split-nitrogen management mainly affects the plant nitrogen concentration at the later growth stage. Similarly, in our study, N3 treatment significantly increased the nitrogen concentration of wheat plants at the later growth stage. Moreover, the enhanced nitrogen use efficiency obtained by optimizing the splitting of nitrogen fertilizer (N3) may be attributed to the fact that the demands of wheat for nitrogen were appropriately met by the timely application of this element, which is an important reason for optimizing nitrogen use^32^. In this regards, Shi et al. found that excessive nitrogen application increased water consumption due to excessive vegetative growth and higher Tr, thereby rendering the wheat prone to drought stress^33^. Our research indicated that the N3 treatment significantly increased water use efficiency. Whereas in contrast, sufficient irrigation decreased water use efficiency, but did not significantly increased grain yield. We suspect that these results can be explained by the fact that the excessive application of water delays maturity, and thus photoassimilates were not converted into grain yield at maturity^34^. This accordingly illustrates that consuming less to produce more is a promising paradigm for the sustainability of modern agricultural production^35^. In conclusion, the optimized regime comprising a 5:5 basal-top-dressing nitrogen ratio and water-saving irrigation can be used increase grain yield and nitrogen use efficiency. Moreover, this treatment was more effective in enhancing water use efficiency than each of the other treatments evaluated. However, we only investigated the responses in a single wheat and same ecological regions. In order to better evaluate the irrigation and split-nitrogen management measures, further research is needed to understand the mechanism in which irrigation and split-nitrogen interacts for higher grain yield and higher resource use efficiency across different wheat varieties and ecological regions.

In the present study, we also detected a significant positive correlation between grain yield and biomass at the grain-filling stage, and a higher grain-filling rate was observed to be beneficial to high yield. In this regard, our investigation of the correlation between photosynthetic parameters and grain sink capacity revealed that grain yield, nitrogen use efficiency and water use efficiency were significantly correlated with photosynthetic and grain-filling parameters^36^. The photosynthetic capacity and grain-filling rate of flag leaves after wheat anthesis are the main factors affecting grain yield. Thus, improving photosynthetic performance of the flag leaf and grain storage capacity of wheat after anthesis through ensuring a rational water-nitrogen interaction is of considerable significance with respect to achieving a high yield and high efficiency. Although, the photosynthetic performance and grain-filling rate of wheat is also influenced by wheat variety. In the present study, we only investigated the responses in a single wheat variety, and thus cultivar-related differences warrant further study in the future.

## Conclusion

To summarize, in the present study, we examined the effects of split-nitrogen fertilizer application and supplemental irrigation on the photosynthetic capacity, grain-filling, grain yield, nitrogen use efficiency and water use efficiency of wheat. Collectively, our findings confirm that under the condition of 240 kg ha^−1^ total nitrogen application, treatments based on a combined regime of water-saving irrigation and a 5:5 basal-top-dressing nitrogen ratio significantly increased the Pn, Tr, IWUE, Fv/Fm, ΦPSǁ, Qp and ETR and decreased the Ci of flag leaves after anthesis, which in turn promoted Vmean and increased the 1000-grains weight. This treatment regime resulted in a significant enhancement photosynthetic capacity of flag leaves and increased grain-filling, and consequently results in the highest grain yield and nitrogen use efficiency and water use efficiency. Therefore, we conclude that treatments based on water-saving irrigation and a 5:5 basal-top-dressing nitrogen ratio are optimal under the experimental conditions we assessed.

## Materials and methods

### Site description

Field experiments were conducted during the winter wheat growing seasons in 2016 and 2017 at the experimental station in Shijiawangzi Village, Xiaomeng Town, Yanzhou, Jining City, Shandong Province, China (35°40′N, 116°41′E). This region is a typical plain region of the NCP, which has a warm temperature continental climate. The soil in the region is classified as a loam soil. Data relating to mean monthly precipitation and air temperature (obtained from the Ji’ning Meteorological Station, China Meteorological Administration) during the entire wheat growth period are shown in Fig. 1. Prior to the experiment, soil properties in the top 0–20 cm layer of soil at the trial site were as follows: organic matter concentration, 14.20 g kg^−1^; total nitrogen, 1.13 g kg^−1^; available nitrogen, 122.60 mg kg^−1^; available potassium, 129.44 mg kg^−1^; and available phosphorus, 38.11 mg kg^−1^. The crop previously grown on the site was corn and all the residual straw was returned to the soil after harvest.

### Experimental design and crop management

The winter wheat cultivar ‘Jimai22’, which is the most commonly planted cultivar in the NCP region, was used as the experimental material. The experiment was carried out based on a randomized completely block design with a split plot arrangement, each with three replications. All plots were 20 m^2^ in size. The distance between two adjacent plots receiving different application treatments was 2 m to avoid the interference of nitrogen and irrigation. We established a water-saving irrigation regime (SI: relative soil water content was maintained up to 70% at the joining and anthesis stages of wheat) and sufficient irrigation (UI: relative soil water content was maintained up to 90% at the joining and anthesis stages of wheat) was used as a control condition. The split-nitrogen regime examined contained a similar total amount of (240 kg ha^−1^) but was split into four different proportions between sowing and the jointing stage; i.e. 10:0 (N1), 7:3 (N2), 5:5 (N3) and 3:7 (N4).

The irrigation amounts is calculated according to the formula: *M* = *10* × *r* × *H* × (*βi* − *βj*). *M* stands for the amount of irrigation. *r* represents the bulk density of the quasi-moist soil depth.* H* stands for the pseudo-moist soil depth. *βi* stands for design water content. And the *βj* is the water content of the soil before irrigation. Irrigate with hose and measure irrigation by meter^6^.

Urea (N 46%), calcium superphosphate (P_2_O_5_ 12%) and potassium chloride (K_2_O 60%) were selected as nitrogen, phosphate and potassium fertilizers, respectively. The amount of phosphate and potash fertilizers were 150 kg ha^−1^ and 112.5 kg ha^−1^ respectively. The basic nitrogen fertilizer and all the phosphate and potash fertilizers spread on the soil surface prior to sowing. Immediately after application, the fertilizer was subsequently mixed into the top 0–20 cm layer of soil using a rotary cultivator. At the joining stage of wheat, nitrogen fertilizer was applied to furrows, which were immediately covered with soil. After fertilizer application, wheat seeds were sown at a depth of 3–5 cm. Sowing on 12th October in 2016 and 24th October in 2017, the three-leaf basic determining was 1.8 million ha^−1^. The receiving dates were 8th June in 2017 and 7th June in 2018. Management of the wheat crop was consistent with the local conventional high-yield cultivation management practices.

### Sample collection, analyses, and calculations

#### Photosynthetic parameters

The net photosynthetic rate (Pn), transpiration rate (Tr), and intercellular carbon dioxide concentrations (Ci) of winter wheat leaves at the filling stage were measured from 09:00 to 11:00 AM on sunny and windless days using a CIRAS-2 Photosynthesis System (PP Systems Company, Britain). Three randomly selected flag leaves per plot were used for the measurement of functional leaf photosynthetic parameters every 7 days in the period from anthesis to maturity (measuring 5 times altogether, every year from 2016 to 2018)^35^. The instantaneous water use efficiency (IWUE) of the flag leaves was measured as the ratio between the net photosynthetic rate and transpiration rate at leaf level^36^.

#### Chlorophyll fluorescence parameters

Chlorophyll fluorescence parameters of flag leaves at the grain filling stage were measured (measuring 5 times altogether, every year from 2016 to 2018) using a FMS-2 pulse-modulated fluorometer (Hansatech-instruments, UK). Three randomly selected flag leaves from each plot were measured, and all measurements were performed on sunny and windless days between 09:00 AM and 12:00 PM. Clamps were used initially to orient the wheat leaves towards the sun before measurement, so that individual leaves at different positions received a consistent amount of light, in the absence of the shading effects of other leaves. Prior to taking measurements of dark-adapted fluorescence parameters, the leaves were fully dark-adapted for 30 min^37^. The maximal photochemical efficiency (Fv/Fm), actual photochemical efficiency (ΦPSǁ), photochemical quenching coefficient and electron transport rate (ETR) were calculated according to Zivcak et al.^38^. In the present study, the reported chlorophyll fluorescence parameter values are the average values of five measurements performed during the grain-filling period.

#### Grain potential storage capacity (GPSC) and storage capacity index (SCI)

Ten randomly selected spikes were harvested from each plot at maturity. All grains were artificially peeled out from each spike. The number of fresh grains was recorded. The grain volume was measured using the drainage method, and the volume of single fresh grain was calculated as grain storage capacity (GSC)^39^.

$$ \begin{aligned} {\text{GPSC}} & = {\text{SPUA}} \times {\text{NOS}} \times {\text{GSC}}; \\ {\text{SCI}} & = {1}000 - {\text{grain}}\;{\text{weight}}/\left( {{\text{GSC}} \times {1}000} \right); \\ \end{aligned} $$

where SPUA is the spike per unit area and NOS is the number of spikes.

#### Grain-filling process

According to the method of Yan et al.^40^, we selected wheat stems at anthesis on the same date and marked these as single stems with similar growth. In each plot 300 single stems were marked to ensure uniformity. Each spikes was sampled from flowering to harvest, and 10 randomly labeled spikes were collected between 09:00 and 11:00 AM at 7-day intervals (sampling 5 times altogether, every year from 2016 to 2018). All harvested spikes were placed in marked paper bags, dried in an oven at 105 °C for 30 min, and then at 75 °C until a constant weight was obtained. Thereafter, the grain was gently threshed from the ears by hand and the material was carefully divided into the grains and the other parts of the ear. The grains were evenly mixed, and subsequently 1000 grains were randomly counted using a manual method, and weighed three times^41^. The grain-filling process was assessed by fitting using the Logistic growth equation^40^. In analysis, we adopted the following secondary parameters to describe the filling characteristics: Tmax (the time to reach the maximum filling rate), Vmax (maximum filling rate), Vmean (mean filling rate) and D (grain filling duration).

#### Nitrogen concentration

Five representative wheat randomly samples were obtained from each plot at before-winter, jointing, anthesis and maturity stages. These samples were oven-dried at 105 °C for 30 min and then at 80 °C to constant mass and then weighed to determine the biomass. All samples were crushed using a ball mill before nutrient analysis. Nitrogen concentrations were determined by the Liu method^42^.

#### Grain yield (GY), water use efficiency (WUE) and nitrogen use efficiency (NUE)

At the maturity stage, grain yield was measured from an area of 2 m^2^ in each plot. To determine soil water consumption during the wheat growing season, the soil water content was measured at sowing and maturity. According to actual circumstances during the experiments, we assumed that the contributions of groundwater recharge, runoff and deep seepage were negligible. The crop water consumption (ET) was calculated using the following soil water balance equation^43^:

$$ {\text{ET }}\left( {{\text{mm}}} \right) = {\text{SWC}} + {\text{rainfall}} + {\text{irrigation}}; $$

WUE was calculated as follows:

$$ {\text{WUE}} = {\text{GY}}/{\text{ET}}; $$

NUE was calculated as follows^44^:

$$ {\text{NUE}} = {\text{GY}}/{\text{NG}}; $$

where NG is the grain nitrogen accumulation.

### Statistical analysis

Correlations between photosynthetic performance parameters and grain filling after anthesis were analysed statistically using SPSS 13.0 software (α = 0.05). The effect of irrigation and nitrogen fertilizer treatments on photosynthetic performance and grain-filling parameters were analysed with an analysis of variance using GLM in SPSS 13.0. A logistics equation of the grain filling was modeled using SPSS 13.0. All charts were produced using Excel and Sigmaplot 12.5 software.

## Data Availability

All data generated or analyzed during this study are included in this published article.
